# Meetings of Societies

**Published:** 1949-01

**Authors:** 


### Meetings of Societies

### BRISTOL MEDICO-CHIRURGICAL SOCIETY

THE Annual General Meeting was held at the University on Wednesday, 13th October, 1948. The retiring President proposed from the chair the election of Professor J. A. Nixon as an Honorary Member. He reminded members how seldom this honour was bestowed, and how much the Society was indebted to Professor Nixon for his work on the staff of the *Journal* during thirty-nine years, for twenty-two of which he was Editor: these years included the difficult period of the last war. The proposal was carried by acclamation.

The new President, Professor H. J. Drew Smythe, then delivered his Presidential Address, published on page 1 of this issue.

The annual reports of the honorary secretary, honorary treasurer and editor were read and approved, and the first two were thanked for their labours during the past year and re-elected. Three members were elected to vacancies on the Committee, and other officers as shown below :

*Committee*, 1948–49

*President :* Professor H. J. Drew Smythe. *President-Elect :* Dr. Orr-Ewing. *Honorary Treasurer :* Dr. Sutton. *Honorary Secretary :* Mr. Eyre-Brook.

*Editor of " Journal " :* Mr. Watson-Williams. *Assistant Editor :* Dr. N. S. Craig (*ex-officio*).

*Honorary Medical Librarian and Representatives on Library Committee of the University :* Professors Perry, Milnes Walker and Drew Smythe.

*Elected Members :* Mr. R. V. Cooke, Mr. A. Palin, Mr. Elwin Harris, Dr. Rowley, Dr. Lennox Robertson, Professor Milnes Walker, Dr. Kersley, Dr. V. Tryon, Dr. Cornall.

### HONORARY SECRETARY'S REPORT

During the year 1947–48 there were sixty-three new members elected, bringing the total membership to 351. We have lost two valued members through death—Mr. Carwardine and Mr. Clifford Moore.

The President (Dr. T. Milling) gave an exceedingly interesting address on " The Life of Edward Jenner ", illustrated by material he had collected from the Welcome Institute and from his own visits to Berkeley, where Jenner lived and worked. The Clinical Meeting was again held in the form of demonstrations, but this year at the Royal Fort instead of the Bristol Royal Infirmary, as formerly. Distinguished visitors who addressed the Society were Professor W. W. D. Thompson and Mr. J. J. Mason Brown. Papers were contributed by Dr. Coralie Rendle Short, Dr. Malcolm Campbell, Dr. Clarke and Dr. Naish, Professor Rendle Short, Dr. Gibson and Dr. Norman.

During the course of the year Professor Nixon resigned the Editorship of the *Bristol Medico-Chirurgical Journal.* The Committee of the Society were delighted to obtain the services of Mr. Watson-Williams as the new Editor.. I am sure we are all delighted to welcome him and none will be ignorant of the many years of hard work that he has put into the *Journal,* both as Assistant Editor and as Editorial Secretary.

### BRISTOL MEDICO-CHIRURGICAL SOCIETY

### INCOME AND EXPENDITURE ACCOUNT for the year ended 30th September, 1946

### EXPENDITURE

| | £ | s. | d. | £ | s. | d. |
|---|---|---|---|---|---|---|
| To Subscriptions to *Medico-Chirurgical Journal* (255 at 10s. 6d.) | | | | 133 | 17 | 6 |
| ,, Grant to Medical War Relief Fund .. .. .. .. .. .. | | | | 250 | 0 | 0 |
| ,, Librarian, Honorarium .. .. | | | | 22 | 10 | 0 |
| ,, Secretary and late Secretary, Honorarium .. .. .. .. | | | | 20 | 10 | 0 |
| ,, Audit Fee .. .. .. .. .. | | | | 4 | 4 | 0 |
| ,, Printing and Stationery .. .. | | | | 23 | 10 | 10 |
| ,, Lewis's Medical and Scientific Library .. .. .. .. .. | | | | 6 | 7 | 6 |
| ,, Catering .. .. .. | 49 | 18 | 9 | | | |
| ,, Other expenses of Meetings .. .. | 15 | 6 | 10 | | | |
| ,, Postages and Sundries | 24 | 18 | 4 | | | |
| | | | | 90 | 3 | 11 |
| | | | | £551 | 3 | 9 |

### INCOME

| | £ | s. | d. |
|---|---|---|---|
| By Members' Subscriptions.. .. | 237 | 1 | 0 |
| ,, Interest on Bank Deposit Account .. .. .. .. .. | 1 | 2 | 4 |
| ,, Interest on £500 4 per cent. Consolidated Stock, *less* Tax .. | 10 | 10 | 0 |
| ,, Deficiency for the Year .. | 302 | 10 | 5 |
| | £551 | 3 | 9 |

### BALANCE SHEET as at 30th September, 1946

### LIABILITIES

| | £ | s. | d. | £ | s. | d. |
|---|---|---|---|---|---|---|
| Sundry Creditors .. .. | | | | 26 | 8 | 0 |
| Due to *Journal* for 1945, on Account .. .. | | | | 9 | 13 | 11 |
| Subscriptions paid in Advance .. .. .. | | | | 3 | 3 | 0 |
| Capital as at 30th Sept., 1945 .. .. .. | 1,130 | 4 | 8 | | | |
| *Less* Deficiency for the year .. .. .. .. | 302 | 10 | 5 | | | |
| | | | | 827 | 14 | 3 |
| | | | | £866 | 19 | 2 |

### ASSETS

| | £ | s. | d. | £ | s. | d. |
|---|---|---|---|---|---|---|
| Cash in Hand .. .. | 3 | 3 | 0 | | | |
| Cash at Bank— | | | | | | |
| Current Account .. | 195 | 19 | 6 | | | |
| Deposit Account .. | 190 | 9 | 8 | | | |
| | | | | 389 | 12 | 2 |
| £500 4 per cent. Consolidated Stock at cost .. .. .. .. | | | | 437 | 9 | [illegible] |
| Arrears of Subscriptions .. .. | | | | 39 | 18 | 0 |
| | | | | £866 | 19 | 2 |

Audited and found correct,
H. E. KEELER,
*Chartered Accountant,*
*Auditor.*

16–18 Clare Street, Bristol 1.
*October,* 1946.

In the Winter issue for 1946 (p. 154) it was announced that the Treasurer's Report would appear in next issue: by an oversight it was omitted. The Accounts and Balance Sheet of the Society are therefore given here.

### BRISTOL MEDICO-CHIRURGICAL SOCIETY

### INCOME AND EXPENDITURE ACCOUNT for the year ended 30th September, 1948

| EXPENDITURE | £ | s. | d. | INCOME | £ | s. | d. |
|---|---|---|---|---|---|---|---|
| To Subscriptions to *Medico-Chirurgical Journal* handed over .. | 356 | 0 | 0 | By Members' Subscriptions.. .. | 560 | 3 | 6 |
| ,, Grant to Medical Library .. | 25 | 0 | 0 | ,, Interest on Bank Deposit Account .. .. .. .. .. | | 14 | 2 |
| ,, Librarian, Honorarium .. .. | 15 | 0 | 0 | ,, Interest on £500 4 per cent. Consolidated Stock, *less* Tax .. | 11 | 0 | 0 |
| ,, Secretary .. .. .. .. .. .. | 10 | 0 | 0 | ,, Deficiency for the year .. .. | 46 | 13 | 7 |
| ,, Audit Fee .. .. .. .. .. .. | 8 | 8 | 0 | | | | |
| ,, Printing and Stationery .. .. | 61 | 5 | 2 | | | | |
| ,, Lewis's Medical and Scientific Library .. .. .. .. .. .. | 8 | 2 | 3 | | | | |
| ,, Catering .. .. .. .. .. .. | 98 | 4 | 3 | | | | |
| ,, Other Expenses of Meetings .. | 18 | 16 | 11 | | | | |
| ,, Postages and Sundries .. .. | 17 | 14 | 8 | | | | |
| | £618 | 11 | 3 | | £618 | 11 | 3 |

### BALANCE SHEET as at 30th September, 1948

| LIABILITIES | £ | s. | d. | £ | s. | d. | ASSETS | £ | s. | d. | £ | s. | d. |
|---|---|---|---|---|---|---|---|---|---|---|---|---|---|
| Sundry Creditors .. .. .. .. .. | | | | 27 | 9 | 6 | Cash at Bank— | | | | | | |
| Subscriptions paid in advance .. | | | | 16 | 16 | 0 | Current Account.. .. | 14 | 6 | 8 | | | |
| Cash due to the *Journal* .. .. | | | | 69 | 16 | 11 | Deposit Account.. .. | 91 | 8 | 6 | | | |
| Accumulated Fund as at 30th Sept., 1947 .. .. | 640 | 15 | 11 | | | | | | | | 105 | 15 | 2 |
| *Less* Deficiency for the year .. .. .. .. .. | 46 | 13 | 7 | | | | Petty cash in hand .. .. .. | | | | 10 | 3 | 1 |
| | | | | 594 | 2 | 4 | £500 4 per cent. Consolidated Stock at cost .. .. .. .. .. | | | | 437 | 9 | 0 |
| | | | | | | | Arrears of Subscriptions .. .. | | | | 154 | 17 | 6 |
| | | | | £708 | 4 | 9 | | | | | £708 | 4 | 9 |

Audited and found correct,

H. E. KEELER,

*Chartered Accountant,*

*Auditor.*

16–18 Clare Street, Bristol 1.

*October*, 1948.

The second meeting of the session was held at the University on Wednesday, 10th November. Professor A. V. Neale read a paper entitled : " **Early Life** ", which we hope to publish in our next issue.

A proposal was submitted for borrowing books from the Library by post, for the convenience of members living out of Bristol, and approved.

The third meeting was a clinical meeting at the University on Wednesday, 8th December. Professor Milnes Walker, " **Coarctation of the Aorta** " ; Dr. A. M. G. Campbell, " **Neurological Cases** " ; Mr. R. V. Cooke, " **Patients Surviving several years after Gastrectomy for Carcinoma**" (Mr. A. W. Adams showed another case); the Streptomycin Research Team, " **Results of Treatment of Tuberculosis Meningitis** " ; Dr. J. M. Naish, " **Hemi–Parkinsonism** " ; Mr. A. L. Eyre-Brook, " **Repair of Cut Flexor Tendons** ".

The President presented the new edition of Laws and Bye-Laws, which were approved by the Society.

The fourth meeting was held at the University on Wednesday 12th January, 1949. Professor Milnes Walker gave an address on " **Surgery in Vascular Disease** ", illustrated by lantern slides, which we hope to publish shortly. The meeting confirmed the alterations in the Laws approved at the previous meeting, and passed a vote of thanks to Mr. Watson Williams for the work he had done in the revision.

---

### DEVON AND EXETER MEDICO-CHIRURGICAL SOCIETY

ON 16th December Dr. F. M. R. Walshe gave an address on "**The Clinical Approach in Neurology.**" *v. B.M.J.*, 1949, i, p. 27.

---

### PLYMOUTH MEDICAL SOCIETY

ON 11th March : Mr. Lambert Rogers (*subject to be announced*).

---

### BRISTOL MEDICAL ASSOCIATION (BATH, BRISTOL AND SOMERSET BRANCH)

THE annual meeting of the above Branch was held at Taunton on 25th November. Mr. J. R. Nicholson-Lailey delivered his Presidential Address entitled : "**Some Points in the History of Taunton and Somerset.**" The officers for the year are : President, Mr. J. R. Nicholson-Lailey, F.R.C.S. ; President-Elect, Dr. Percy Phillips ; Vice-Presidents, Dr. R. G. Gordon and Dr. H. J. Orr-Ewing ; Hon. Secretary, Dr. O. E. L. Sampson ; Hon. Treasurer, Dr. C. H. G. Price.

A meeting of the branch was held at Musgrove Park Hospital, Taunton, on Saturday 29th January. Mr. J. Barron. F.R.C.S. gave an address on **"Plastic Surgery."**

The main item of interest in the Branch Council report was the comment on the extremely successful meeting held at Bath in February, 1948. Mr. Justice Birkett addressed in a most interesting and amusing manner a record attendance of 270 members.

Forthcoming meetings: The meeting scheduled for 23rd February at Bath will be held on 16th February instead.

A clinical meeting is being arranged at the Bristol Royal Infirmary for 30th March.

---

### BRITISH MEDICAL ASSOCIATION (BRISTOL DIVISION)

A GENERAL meeting of the Division was held at the University on Wednesday, 17th November. It was devoted to a film show: "Patent Ductus Arteriosus; Anterior Poliomyelitis; Pathé News Reel of Southmead Quadruplets."

On 27th April Professor Henry Cohen will deliver a lecture in the large physics theatre (Royal Fort). The subject will be announced later.

---

### THE SOUTH-WESTERN LARYNGOLOGICAL ASSOCIATION

THE first meeting of the session was held on 2nd October, 1948, at the Gloucester Royal Infirmary, Mr. Watson-Williams in the chair. Eleven members and three visitors were present. A number of interesting cases were shown by Messrs. Gibb, Barker and Mower: notably, brother and sister with hereditary syndactyly and scleroderma, and œsophageal stricture: and several cases illustrating results obtained by radiotherapy.

The second meeting of the session was held on 8th January at the Royal Gwent Hospital, Newport. Messrs. Sutton and J. L. D. Williams showed several series of cases: (*a*) after operation for brain abscess; (*b*) bone complications of frontal sinusitis; (*c*) maxillary osteomyelitis in infants; (*d*) keratosis laryngis; (*e*) deafness after meningococcal meningitis; and a number of others. A lively discussion took place.

The next meeting of the Association will be on 2nd April, at Bournemouth.

